# Event-Related Brain Potential Correlates of Event-Based Prospective Memory in Children With Learning Disability

**DOI:** 10.3389/fpsyt.2022.898536

**Published:** 2022-06-22

**Authors:** Lili Ji, Qi Zhao, Yafei Zhang, Jiaojiao Wan, Yifan Yu, Junfeng Zhao, Xiaoming Li

**Affiliations:** ^1^Institute of Behavior and Psychology, School of Psychology, Henan University, Kaifeng, China; ^2^Department of Psychology, Faculty of Social Sciences, University of Macau, Macau, Macau SAR, China; ^3^Center for Cognitive and Brain Sciences, University of Macau, Macau, Macau SAR, China; ^4^Department of Health Promotion, Education, and Behavior, University of South Carolina, Columbia, SC, United States

**Keywords:** children with learning disability, event-based prospective memory, event-related potentials, N300, prospective positivity

## Abstract

Prospective memory (PM) has been reported to be impaired in children with learning disabilities (LD), but few studies have examined the underlying neural mechanism of this impairment. To address this issue, the present study applied ERP technique to explore the difference of event-based prospective memory (EBPM) in 21 children with LD and 20 non-LD children with double task paradigm. Results from behavioral data showed that LD children exhibited lower accuracy than non-LD children. The ERP results showed that the two groups displayed significant difference in the ERP components, with longer N300 latency in LD group, but there was no obvious difference found in the prospective positivity component. The present findings seem to indicate that the poor performance of LD children on PM task might be result from deficits in PM cues detection. These results provided evidence for the existence of altered PM processing in LD children, which was characterized by a selective deficit in cues detection of PM. Therefore, these findings shed new light on the neurophysiological processes underlying PM in children with LD.

## Introduction

Many children have suffered problems in learning, with 17.9% of them including combined reading and mathematical disabilities (1). Learning disability (LD) is usually characterized by multiple cognitive weaknesses, probably resulting from neurobiological etiology and variations in brain development (2, 3). Specifically, previous studies have indicated that LD children showed deficits in some sorts of cognitive domains, involvement memory, attention, executive function and so on (4, 5). As an important aspect of episodic memory, prospective memory (PM) plays crucial role in the learning and daily life of LD children, but it is also reported being impaired among these children (6–8).

PM refers to the memory of carrying out preplanned events and activities at the appropriate situation or some future point in time (9, 10). For example, remembering to bring a pass before taking a school exam (event-based prospective memory, EBPM) or going to the hospital for making an appointment with the doctor at 5 pm (time-based prospective memory, TBPM). It's believed that the cognitive process of underlying PM performance includes four stages, namely intention formation, intention maintenance, intention initiation and intention execution (11, 12). Cue identification and intention retrieval are two important components underlying the intention initiation stage of PM (13). Cue identification refers to the detection of clues needed to perform certain activities. Intention retrieval involves the recalling of what kind of intentional behavior should be implemented (13, 14). Modern cognitive neuroscience and clinical research had provided growing and important evidence for the study of the neural mechanism of PM, especially EBPM. Moreover, researches using event-related potentials (ERPs) have displayed that two ERP components, N300 and prospective positivity (PP), were differentially related to the cue identification and intention retrieval of PM in humans (15–17). N300 is a negative wave in the occipital-parietal region at 300–500 ms after the presentation of PM target cues, which reflects the individual's detection of PM target cues (18–21). N300 is very similar to N2 in duration and morphology. West (15) discussed the similarities and differences between N300 and N2 in study (15). They found that N300 only appears in prospective memory task and reflects the awareness of target cue in PM task. PP is widely distributed in the central, parietal and occipital regions at 400–1,200 ms after the appearance of PM target cues, associated with the process of intentional retrieval of PM (18, 19, 21, 22). PP are often confused with two other temporally distinct components of the ERPs. One is the detection of low probability targets (P3), the other is the recognition of PM cues (parietal old-new effect). Similarities of distribution and morphology in brain regions among the PP, P3 and recognition old-new effect lead one to wonder whether these are in fact the same components observed in different paradigms. However, evidence from a number of studies suggests that the P3 and parietal old-new effect can be distinguished from the PP (23–25). West and Krompinger (23) investigated the neural basis of PM, and found that the PP was only induced in the PM task, but not in the recognition task and the cue recall task. These evidences further confirmed that N300 and PP were two specific wave components of PM (26).

Some scholars have also carried out cognitive neuroscience and clinical research for special groups such as Schizophrenia patients, Attention Deficit children, Alzheimer's patients, Alcohol Dependency Syndrome patients, and Medial Temporal Lobe Epilepsy patients. These studies found that different PM impairment may be caused by different brain function damage and neurological deficits (27, 28). PM deficits in LD children have also been drawing rising attention over the past decade. A large number of studies have indicated that executive functions are assumed to be involved in PM (29–33). Usually, PM requires the integration of several complex processes, and successful PM requires a certain level of executive functioning ability, including updating, inhibition, shifting. Updating abilities maintain the intention of PM (34, 35). Shifting is mainly reflected in the ability to involve shifting between two tasks (ongoing and PM tasks) when PM cues appeared to be a key factor in the successful implementation of PM (35). Similarly, inhibition is necessary when a habitual dominant action must be inhibited and replaced by a new one (36). Therefore, impairments in these aspects of executive functioning may be relevant for failure of PM performance (37). Unfortunately, a substantial number of studies showed students with LDs have problems in executive function (38–42). Moreover, some studies have shown that PM is significantly related to LD. Compared to non-LD children, LD children may report more PM problems, or at least in some cases, the PM performance of LD children was far worse (6–8, 43). But up to now, only a few behavioral studies are focused on PM performance of LD children and some of them still have inconsistent results, including underlying factors of PM weak ability. Dong et al. (43) showed that the performance of LD children was as good as that of typical students in EBPM tasks but not as good as that of typical students in time-based PM tasks without reminders. Chen et al. (7, 8) found that high achieving students outperformed low achieving students in EBPM tasks. Zhang et al. (6) adopted a multinomial modeling approach to study the EBPM in LD children, indicating that prospective components in LD children were lower than control group, while no significant difference was observed in retrospective components.

Then, it can be seen that the characteristics of PM deficits in LD children is still unclear. Moreover, most of existing studies on LD children are explored from behavioral perspectives and little is known about the underlying cognitive and neural mechanisms of PM in LD children with ERP. Considering PM playing an important role in LD children's learning and normal life as well as addressing the knowledge gaps, in the current study we applied an event-based PM paradigm with ERPs to investigate PM abilities in LD children. Based on previous studies, we not only paid attention to behavioral measures, but also adopted ERP technique to study whether LD children would show different patterns during EBPM compared to non-LD controls and whether both PM cue detection (N300) and PM intention retrieval (PP) components may be impaired.

## Materials and Methods

### Participants

The 21 children with LD (13 boys, age: 12.38 ± 0.56 years) and the 20 age-matched non-LD children (11 boys, age: 12.16 ± 0.62 years) were selected from one middle school (Grades 7–9) in Kaifeng, Henan province, China.

In the current study, we applied a rigorous method to screen the prospective participants. First, all the 538 students in the school were tested with Learning Adaptability Test (AAT) (44). The 68 students receiving level 2 or below on AAT scores were selected. Second, we invited the head teachers to fill in an adapted Chinese version of Pupil Rating Scale (PRS) for 68 students and screened the 53 students who received scores <65 as suspected LD children. Third, we collected students' mathematical and linguistical scores on their latest mid-term and final examinations. Based on the recommendations in the existing studies (45–47), the 22 students who scored <25th percentile in their grade level on these two disciplines were considered as LD children. Finally, one student with low IQ test result based on the Raven Standard Intelligence Test (SPM) (48) were excluded and 21 students were confirmed as the LD group.

All participants (*n* = 21) in the control group met the following eligibility criteria: (1) the SPM score was at the normal level; (2) the score of AAT was above the middle level; (3) the mathematical and linguistical scores in both the latest mid-term and final examinations were >25th percentile in their grade level; (4) matched on intelligence. The EEG data of one subject were deleted due to excessive artifacts, and eventually 20 students were confirmed as the control group.

Before enrolling in the study, all of the children were ensured with right-handed, no color-blindness, and with no previous psychiatric and neurological diseases or emotional disorders. All participants' parents signed written informed consents after they acquainted the procedures thoroughly.

### Study Design

The experiment employed a mixed factorial design with between subjects factor of condition (LD vs. control group) and within-subjects factor of trial (PM vs. ongoing task).

### Stimuli and Event-Based PM Paradigm

We adopted dual task paradigm used in the study of West et al. (49), the ongoing task involved a color discrimination task of phrases. On ongoing activity trials, two colored phrases were presented in the center of a computer screen. The participants' task was to decide whether two phrases were the same color. In the PM task, the participants were required to pause the ongoing task and switch to the PM task by pressing the appropriate button when the same two phrases in coding stages PM target cues which were appeared on the screen again.

The phrases were obtained on the basis of category naming experiments. In the category naming experiment, 27 categories familiar to the subjects were selected (50), and the subjects were asked to list 8 examples for each given category. The samples listed in the first 12 digits of the cumulative frequency were randomly selected from each category to form 648 pairs, which were divided into two groups of 324 pairs each. Half of the phrases were the same color, for example “Word1–Word2 (both words presented in blue)”, the other half were of different colors, for example “Word1 (presented in orange)—Word2 (presented in purple). We used e-prime2.0 programming to ensure a rigorous experimental procedure.

**Figure 1:**
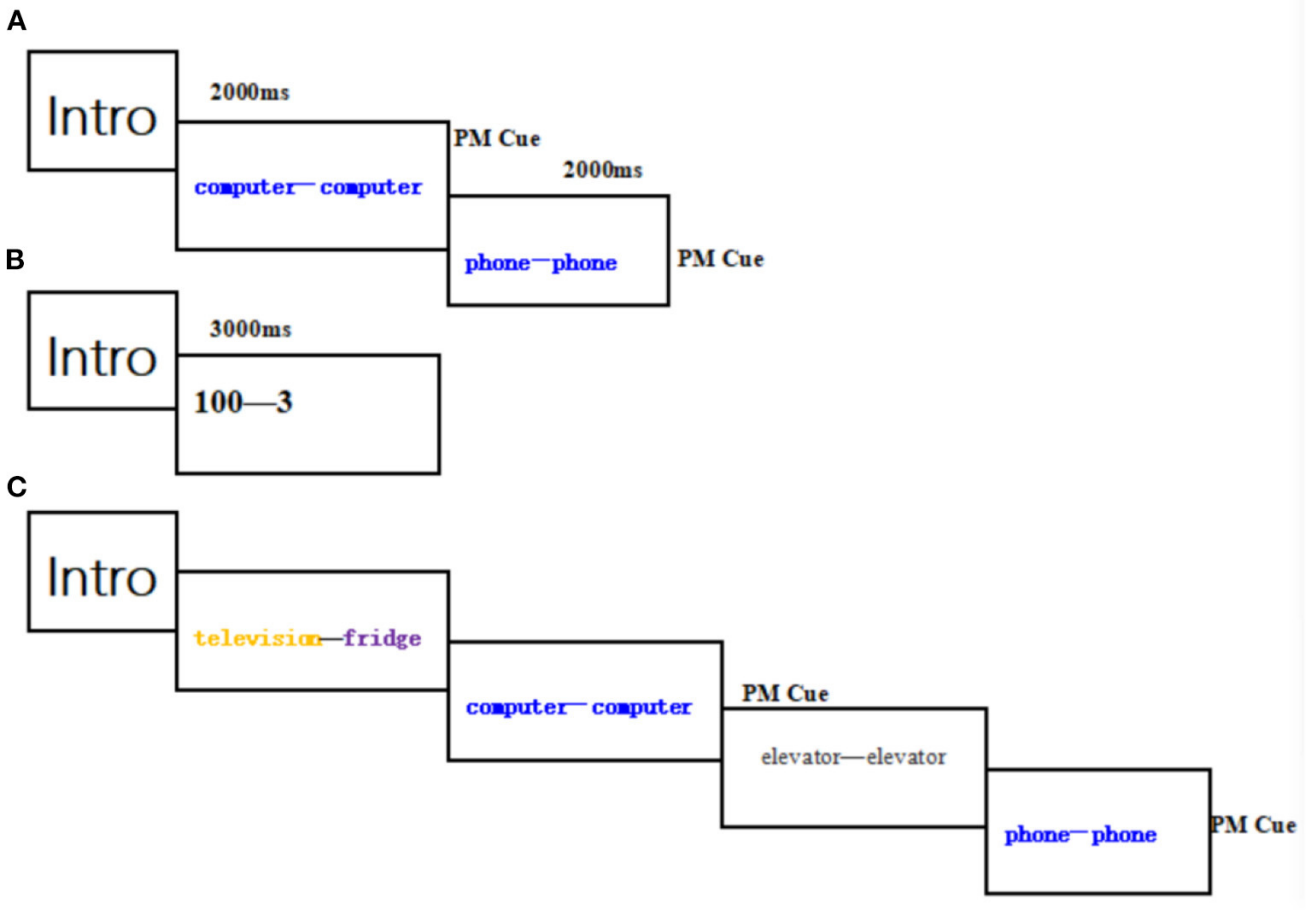
Example block. each block consists of three stage: **(A)** coding stage; **(B)** distraction task stage; **(C)** ongoing task stage.

There were 27 blocks each of which consisted of three stages (Figure 1). In the coding stage: two PM cue trails (each trial consisting of two phrases with the same semantics and color) were presented randomly for 2,000 ms followed by 1,000 ms of blank screen. Participants were asked to remember the two phrases, including their semantics and colors. In the distraction task stage: a three-digit number on the screen after the coding stage lasting 3,000 ms, participants were required to perform “minus three” task for a minute to avoid repeating items they had just learned. In the ongoing task stage each block contained 24 trials which were divided into 22 ongoing activity trails and 2 PM cues trails. Participants would press the “1” key if they judged that the colors of the two words were same and the “2” key if the colors of the two words were different without considering the semantics. However, participants were instructed to press the “0” key if they saw the previous PM target cues again as presented in coding stages on the screen. In each block, PM target cues occurred once in the first 11 trails and once in the last 11 trails. Before the formal experiment, participants are allowed to complete a practice block to ensure that they fully understood the whole experimental procedure. The present study was carried out in according with the guidelines approved by the Institutional Review Board of Henan Provincial Key Laboratory of Psychology and Behavior. All participants in this study gave written informed consent to participate in the experiment.

### ERP Data Recording and Analysis

The ERP data were recorded from a 32-channel Ag/AgCI electrodes cap (Brain Products, Munich, Germany). The electrodes placed at standard locations were arranged according to the extended international 10–20 system. Brain Vision Analyzer 2.0 software (Brain Products, Munich, Germany) was used for off-line analysis. The mastoid of the left ear was used as the reference electrode. The ground electrode was the midpoint on the line connecting FCz and Fz. Electrodes were placed besides the eyes to record the HEOG, and above and below the right eye to record the VEOG. All inter-electrode impedance was maintained below 5 kΩ. The ERP data were recorded using a 0.01–100 Hz bandpass filter and continuously sampled at 500 Hz/channel for offline analysis. Data were first re-referenced to link the left and right mastoids for offline analysis. The low-pass offline filter was 50 Hz. Amplitudes over ± 100 μV were regarded as artifacts and were excluded. The ERP observation window were extracted between the 200 ms pre-stimulus and 1,000 ms post-stimulus time points. The baseline correction was performed in 200 ms pre-stimulus interval. Specifically, data accompanied by artifacts such as bad channels, eye blinks, and eye movements were excluded. The ERPs evoked by PM cue trials and ongoing activity trials were calculated by averaging individual artifact free trials in each participant. Finally, the grand-averaged ERPs were computed and averaged for correctly performed PM cue trials and correctly performed ongoing activity trials in each group.

### Statistical Analysis

For behavioral data, mean accuracy and mean response time (RT) were analyzed for in each task type for PM and ongoing task of two groups. Measurement data were presented as (mean ± standard deviation), and the results showed that the data were normally distributed. Trials with incorrect response or response time faster than 100 ms or slower than 2,000 ms were eliminated. Repeated-measures ANOVAs with task type (PM vs. ongoing task) as a within-subject factor and group (LD vs. control group) as a between-subject factor were performed separately for the accuracy and RT.

With respect to the ERP data, only correct responses and RT in normal range to the stimuli were included in ERP analyses. Based on the previous ERP studies (16, 17), we analyzed mean amplitude of two ERP components related to PM, namely, N300 and PP, in different task conditions. Specifically, according to the relevant literature (51, 52), the peak amplitude and latency of N300 component (300–500 ms) over occipital region (electrodes: O1/Oz/O2) was quantified. The mean amplitude of PP (400–1,000 ms) was quantified at F3, F4, Fz, C3, Cz, C4, P3, Pz, P4. The peak amplitude and latency of N300 and mean amplitude of PP were conducted by using a two-way mixed ANOVA with task type (PM vs. ongoing task) as within-subject factor and group (LD group vs. control group) as the between-subject factor, respectively. For all the analyses in this study, *p* < 0.05 was considered to be statistically significant, and the *p*-values were adjusted using the Greenhouse-Geisser correction when appropriate.

## Results

### Behavioral Performance

#### Accuracy

For the mean accuracy (see Table 1), there was a significant main effect of task type [*F*_(1, 39)_ = 19.112, *p* = 0.000, $\eta_{p}^{2}$ = 0.397] with accuracy being significantly higher for ongoing activity trials than for PM cue trials. Meanwhile, there was a significant main effect of group [*F*_(1, 39)_ = 25.669, *p* = 0.000, $\eta_{p}^{2}$ = 0.338], with the lower accuracy of LD group than that of control group. More importantly, we found a robust interaction between group and task type [*F*_(1, 39)_ = 14.255, *p* = 0.007, $\eta_{p}^{2}$ = 0.268]. An analysis of simple effects showed that this interaction was driven by lower accuracy on PM cue trials in LD group compared with control group and lower accuracy (*p* < 0.001) for PM cue trials than ongoing activity trials in LD group (*p* < 0.001).

**Table 1:** Mean accuracy and RT (M ± SD) of LD group and controls group.

	***N***	**Ongoing task**	**PM**	**F_**GROUP**_ (*p*)**	**F _**TASK**_(*p*)**	F_TASK*GROUP_ (*p*)
**Accuracy**
LD Group	21	0.883 ± 0.104	0.625 ± 0.241	25.669 (0.000***)	19.112 (0.000***)	14.255 (0.007**)
Control Group	20	0.926 ± 0.072	0.888 ± 0.072			
**Reaction time (ms)**
LD Group	21	1,092.002 ± 211.163	1,375.203 ± 212.265	8.872 (0.007**)	33.539 (0.000***)	0.631 (0.537)
Control Group	20	966.051 ± 192.815	1,165.054 ± 230.006			

^**^p < 0.01, ^***^p < 0.001.

#### Reaction Time

For the mean RT (see Table 1), there was a significant main effect of group [*F*_(1, 39)_ = 8.872, *p* = 0.007, $\eta_{p}^{2}$ = 0.167], indicating that LD children performed more slowly than control group. Moreover, there was also a significant main effect of task type [*F*_(1, 39)_ = 33.539, *p* = 0.000, $\eta_{p}^{2}$ = 0.475], with response times being longer for PM cue trials than for ongoing activity trials. However, the interaction between group and task type was not significant (*p* > 0.05).

#### ERP Results

**Figure 2:**
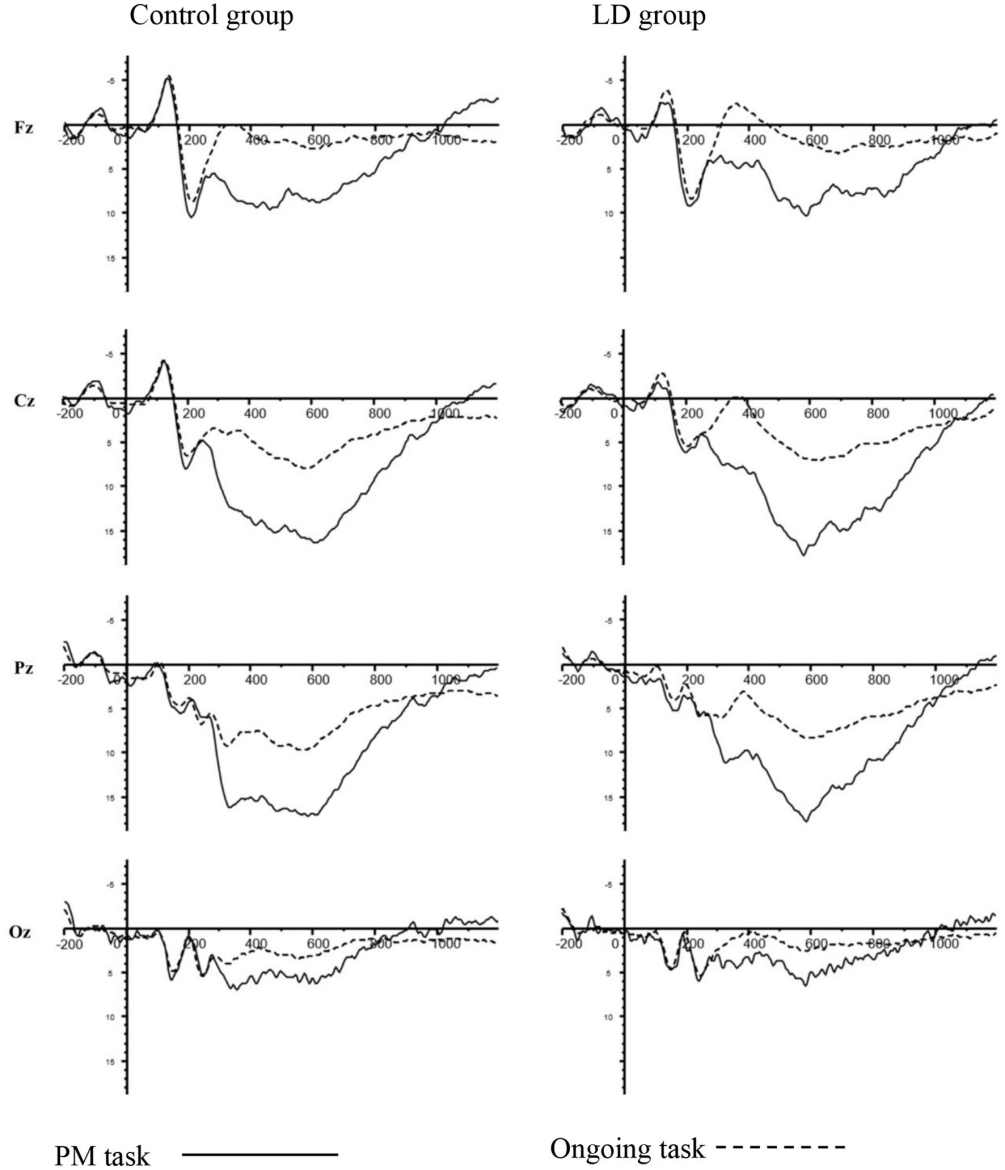
N300 and PP for PM cue trials and ongoing activity trials in LD group and control group at Fz, Cz, Pz, and Oz electrodes.

Figure 2 showed the grand-averaged waveforms of children with LD group and control group. The mean (M) and standard deviation (SD) for each component were displayed in Table 2.

**Table 2:** ERP components (M ± SD) for LD group and control group.

	***N***	**Ongoing task**	**PM**	**F_**GROUP**_ (*p*)**	**F _**TASK**_(*p*)**	
**N300 latency**						**F**_TASK*GROUP*ELECTRODES_ **(*****p*****)**
Total	41	244.433 ± 7.957	282.889 ± 11.114	5.840 (0.020*)	0.005 (0.094)	3.452 (0.04*)
LD group	21	279.873 ± 11.389	285.905 ± 10.616			
Control group	20	248.100 ± 14.954	240.767 ± 10.878			
**PP amplitude**						**F**_TASK*GROUP_ **(** ***p*** **)**
Total	41	7.963 ± 0.497	15.641 ± 0.630	3.150 (0.001**)	170.859 (0.000***)	8.000 (0.084)
LD group	21	7.933 ± 0.694	17.334 ± 0.902			
Control group	20	7.944 ± 0.711	13.949 ± 0.880			

^*^p < 0.05, ^**^p < 0.01, ^***^p < 0.001.

#### N300

For the peak amplitude of N300, there were no significant main effect of group (*p* > 0.05). Meanwhile, the interaction between group and task type was also not significant (*p* > 0.05).

For the latency of N300, there was a significant main effect of group [*F*_(1, 39)_ = 5.840, *p* < 0.05, $\eta_{p}^{2}$ = 0.130], with the latency of N300 being longer in LD group than control group. More importantly, group × task type × electrodes interaction was significant [*F*_(1, 39)_ = 3.452, *p* < 0.05, $\eta_{p}^{2}$ = 0.081]. An analysis of simple effects revealed that this interaction was due to the longer latency of the N300 for PM cue trials in LD group than control group at Oz (*p* < 0.001).

#### PP

Regarding the amplitude of PP, there was a significant main effect of group [*F*_(1, 51)_ = 3.150, *p* < 0.01, $\eta_{p}^{2}$ = 0.075], with eliciting larger amplitude of PP in LD group than control. There was also a significant main effect of task type [*F*_(1, 39)_ = 170.859, *p* < 0.01, $\eta_{p}^{2}$ = 0.814], with an enhanced PP for the PM cue trials than for ongoing activity trials. However, the interaction between group and task type was not significant (*p* > 0.05).

## Discussion

The present study investigated the ability to perform the EBPM task and the neural underpinning in LD children. At the behavioral level, LD children had significant lower accuracy on PM cue trials compared to the control group, indicating that LD group's poor performance may be associated with PM deficits, which replicated results from prior studies (6–8). From the perspective of PM, Chen (8) believed that in most cases LD children performed worse in PM owning to their weaker switching ability between two tasks (53). The completion of PM tasks required attention allocation, transfer and inhibition control (54), but LD children were significantly worse than control group in the aspects of central executive function (55, 56). Therefore, LD children indeed needed to distribute more attentional resources to accomplish PM task. Despite such evidence, the specific neural and cognitive mechanisms of EBPM in LD remain unclear. The ERP data may shed specific insights on it.

As to the ERP results of N300 related to the detection of PM cues, this study found longer N300 latency for PM cue trails in LD group. A number of existing studies suggest that the N300 is sensitive to cue detection of PM cue (15–17, 52). The findings might reflect the impairment in PM cue detection in LD group, indicating more cognitive resources were needed to identify an PM cue from tasks. This result is consistent with previous studies in EBPM of children with LD (6, 57, 58). The significant difference of prospective memory performance between special group and general group was mainly due to preparatory attention processing (prospective component), including LD Children, older people, moderate-to-severe traumatic brain injury and other groups with attention disorders. As mentioned in Mattli et al.'s (59) study, the unsuccessful of attentional strategy monitoring linked to frontally mediated processes of executive attention may lead to the failure of cue identification in PM of students. The physiological basis of prospective memory was related to the prefrontal lobe function, which was mainly responsible for intention-maintenance target monitoring in prospective memory tasks (60). However, LD children may have deficits in attention function of prefrontal lobe function (61–63). It was difficult for LD children to have enough attention resources to complete the preparatory attention processing. Therefore, LD children could not identify distinguish PM cues in time from the ongoing task.

Based on the evidence of the significantly larger activity for the PM cue trials than for ongoing activity trials across two groups, but the non-significant interaction between task type and group on the amplitude of PP, Meanwhile, for the amplitude of PP, we didn't find significant difference across groups in PM cues trails. This might indicate when LD children were able to pay attention to PM cue, and judge the current words as a prospective target, LD children seemed to have the ability to switch tasks between two tasks and retrieve a PM intention from memory. It seemed that LD children with poorer PM performance may not be caused by weak PM intention retrieval ability. Nevertheless, although other studies also had found the similar phenomenon (6, 58), successful PM intention retrievement requires a certain level of executive functioning ability, including shifting, inhibition (64–66). However, a substantial number of studies showed students with LDs had problems in executive function (38–42). Therefore, whether LD children were damaged in the PM intention retrieval was still a key aspect deserving further research. Additionally, PP in LD children showed dramatically larger in the whole tasks than that of control group, indicating that LD children need more cognitive resource in process of PM task and ongoing task and demonstrating cognitive deficit for LD children (67, 68). Taken together, these findings suggested that LD children had the impairment in processes associated with PM cues detection.

Furthermore, ERP results from PP showed larger activation for PM cue trials than ongoing activity trials in both two groups, which was consistent with previous PM studies (15–17). The PP components demonstrated enhanced amplitudes in response to PM cue stimuli, which required a greater recruitment of cognitive resources (69, 70). To some extent, this observed results seemed to support and verified the theory of preparatory attentional processes, which suggested that PM task was a non-spontaneous process, and the monitoring and identification of target cues need to consume more attentional resources. Even if the target cue had not yet appeared, the participants still kept in a promptness state in order to retrieve the PM intention (57, 71).

PM control processing theory supported that in the process of PM, from intention formation to cue detection and intention extraction, it would occupy a large number of cognitive resources and required working memory, especially the participation of central executive system (72). Existing literatures have proved that executive function and PM have similar physiological mechanisms (34, 73, 74). Executive function at different level has different effects on PM (75–77). LD children who always fail to complete the PM task may be due to the deficiency of the central executive function, ultimately leading to poorer PM performance. At present, the predictive effects of executive function components on PM are still inconsistent, and the effects of executive function components on PM differ in term of age (73). Then, for LD children as a special group, which component of central executive function can better predict PM deserves further research.

## Conclusion

The current study revealed that the major deficits in LD children during PM, with overall worse behavioral performance and longer latency of ERP component (N300). These findings might suggest the PM deficits in LD children characterized by a selective deficit in PM cues detection, not the absent of PM intention retrieval. Future research is needed to further confirm these results and to explore the biochemical mechanisms underlying these results.

## Data Availability Statement

The datasets presented in this article are not readily available because the data involving special minority participants must be kept confidential. Requests to access the datasets should be directed to JZ, jfzhao63@hotmail.com.

## Ethics Statement

The studies involving human participants were reviewed and approved by Institutional Review Board at the Henan University in China (IRB 00007212). Written informed consent to participate in this study was provided by the participants' legal guardian/next of kin.

## Author Contributions

LJ and JZ designed the study and drafted the manuscript. LJ, YZ, and JW performed the study. JZ, QZ, and YY analyzed the data and editing of the paper. JZ revised the paper. All authors contributed to the article and approved the submitted version.

## Funding

This work was supported by the Science and Technology Research Project of Henan Provincial Department of Science and Technology [212102310985], the Humanities and Social Science Research Project of Henan Provincial Department of Education [2020-ZDJH-026], and the Social Science Planning Project of Henan Province [2021CJY051].

## Conflict of Interest

The authors declare that the research was conducted in the absence of any commercial or financial relationships that could be construed as a potential conflict of interest.

## Publisher's Note

All claims expressed in this article are solely those of the authors and do not necessarily represent those of their affiliated organizations, or those of the publisher, the editors and the reviewers. Any product that may be evaluated in this article, or claim that may be made by its manufacturer, is not guaranteed or endorsed by the publisher.
